# Association between Serum Vitamin Levels and Depression in U.S. Adults 20 Years or Older Based on National Health and Nutrition Examination Survey 2005–2006

**DOI:** 10.3390/ijerph15061215

**Published:** 2018-06-09

**Authors:** Xiaomin Huang, Yun Fan, Xiumei Han, Zhenyao Huang, Mingming Yu, Yan Zhang, Qiaoqiao Xu, Xiuzhu Li, Xinru Wang, Chuncheng Lu, Yankai Xia

**Affiliations:** 1 State Key Laboratory of Reproductive Medicine, Institute of Toxicology, Nanjing Medical University, 101 Longmian Road, Nanjing 211166, China; huang_minminminmin@163.com (X.H.); yunfan_njmu@163.com (Y.F.); hanxiumei@njmu.edu.cn (X.H.); huangzhenyao@gmail.com (Z.H.); ymm772679723@163.com (M.Y.); zhangyan_njmu@163.com (Y.Z.); 15251751698@163.com (Q.X.); lixiuzhu0808@163.com (X.L.); xrwang@njmu.edu.cn (X.W.); 2 Key Laboratory of Modern Toxicology of Ministry of Education, School of Public Health, Nanjing Medical University, 101 Longmian Road, Nanjing 211166, China

**Keywords:** NHANES, vitamin, depression, vitamin B12, folate

## Abstract

Depression is one of the leading causes of disability around the world. Although several studies have been conducted to analyze the association between vitamins and depression, the results have been inconsistent. Based on the database of National Health and Nutrition Examination Survey (NHANES) (2005–2006), a cross-sectional analysis was conducted to uncover the correlations between serum vitamin concentrations and depression in 2791 participants over 20 years of age. Vitamin concentrations in serum were measured by high performance liquid chromatography (HPLC), a standardized liquid chromatography-tandem mass spectrometry (LC-MS/MS) or radioassay kit method. A nine-item Patient Health Questionnaire (PHQ-9) was used to assess depression symptoms. The binary logistic regression model was applied to analyze the association between vitamins and depression. In the whole population, negative associations were discovered between folate concentrations (*p* for trend = 0.02), *trans*-β-carotene (*p* for trend = 0.01) and depression, while positive associations were found among vitamin B12 concentrations (*p* for trend = 0.008), vitamin A concentrations (*p* for trend = 0.01) and depression. In order to evaluate the influence of gender on the pathogenesis of depression of vitamins exposure, we performed gender-stratified analysis. In females, folate concentrations (*p* for trend = 0.03) and vitamin B12 concentrations (*p* for trend = 0.02) were correlated with depression. In males, no significant association was found between depression and serum vitamin concentrations. The correlation of vitamins with depression deserves further investigation in larger and diverse populations, especially in females.

## 1. Introduction

Depression is not only a common mental disorder that causes people to lose interest in life, experience persistent sadness, and find themselves unable to carry out daily activities; but is also a major mental health problem that represents the biggest share of the world’s burden of disease [1]. World Health Organization (WHO) reported that 322 million people suffered from depression globally and the prevalence of depression around the world was 4.4% (5.1% in females versus 3.6% in males) in 2015 [2]. In the U.S., the prevalence of depression was 4.45% in 2013 [1] and 5.9% in 2015 [2].

Factors contributing to depression include biological, psychological and social environments etc.; among these, diet and nutrition, especially vitamins, largely cause depression. Vitamins are organic compounds that are necessary for reproduction, growth, and the maintenance of the body [3]. There are two forms of vitamins: fat-soluble vitamins; including vitamin A (VA), D (VD), E (VE) and K (VK); and water-soluble vitamins containing vitamin B (VB) and C (VC).

Research has demonstrated that vitamin supplements contribute to a decrease in the risk of depression [4,5,6,7]. The Chicago Health and Aging Project reported that vitamin B6 (VB6) and vitamin B12 (VB12) concentrations were negatively associated with depression, while folate concentrations had no relationship in the elderly [8]. In the SUN cohort study, men with depression had low folate intake, and women in depression had low consumption of VB12, while consumption of VB6 made no significant difference to depression in both genders [9]. A Japanese cross-sectional study concerning the elderly showed that there was no association between vitamin concentrations and depression in men, but there was an inverse relationship between vitamin concentrations and depression in women [10]. Although several studies have investigated the associations between vitamin concentrations and depression, they only focused on representative vitamins or specific age groups. Their results were inconsistent.

In this study, in order to comprehensively assess the correlations between vitamin concentrations and depression, serum concentrations were divided into four groups. The extent of depression was evaluated by the 9-item Patient Health Questionnaire (PHQ-9). In our study, we tried to reveal the relationship between the level of diverse serum vitamins and depression, based on lifespan observation (20-year old and older) using the binary logistic regression model.

## 2. Materials and Methods

### 2.1. Study Populations

**Figure 1:**
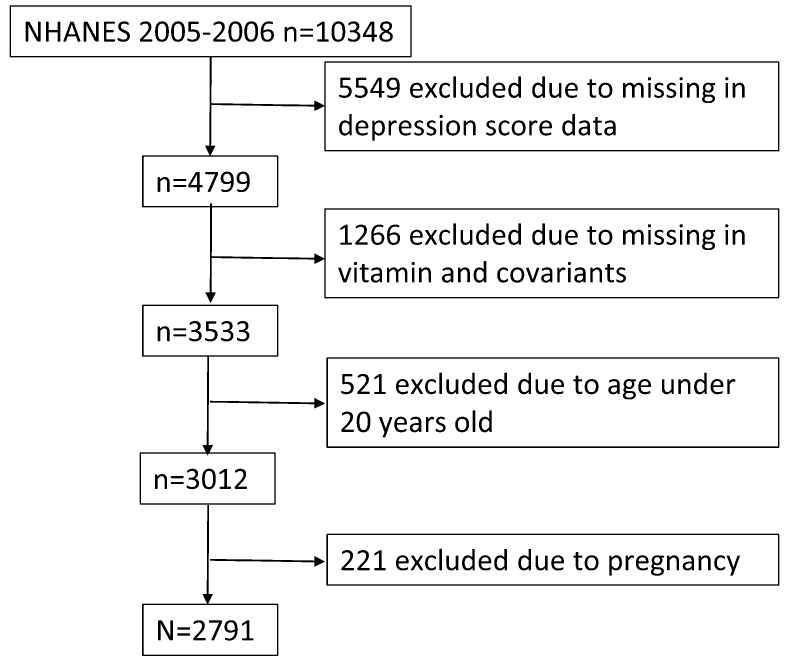
Participant recruitment chart.

Our data was obtained from the National Health and Nutrition Examination Survey (NHANES), a program of the National Center for Health Statistics in US Center for Disease Control and Prevention. The NHANES program is a nationally representative and multistage sampling survey from the 1960s to now. Approval was obtained from the National Center for Health Statistics Ethics Review Board (Protocol # 2005–06). All the participants endorsed informed consent. 10,348 adults and children were eligible for the present study in NHANES 2005–2006. 5549 participants were excluded for a lack of depression score, and 1266 participants were excluded for missing data of level of serum vitamins and covariates. 521 participants under 20 years old were not taken into account due to their different education classifications. 221 pregnancy females were also excluded for their physiological influence. Finally, 2791 participants were included in our study (Figure 1).

### 2.2. Assessment of Serum Vitamins

Serum vitamin concentrations (folate (VB9), 4-pyridoxic acid (PA), pyridoxal 5′-phosphate (PLP), VC, α-carotene (AC), *trans*-β-carotene (*trans*-BC), *cis*-β-carotene (*cis*-BC), b-Cryptoxanthin (BC), γ-Tocopherol (GT), lutein and zeaxanthin (LZ), trans-lycopene (trans-L), retinyl palmitate (RP), retinyl stearate (RS), VA, α-tocopherol (AT), and total (*cis*- and *trans*-) lycopene (TL)] were measured in 100 μL serum by high performance liquid chromatography (HPLC). 25-hydroxyvitamin D (25(OH)D) concentrations were detected by a standardized liquid chromatography-tandem mass spectrometry (LC-MS/MS) method. Serum folate and VB12 concentrations were measured by using the Bio-Rad Laboratories “Quantaphase II Folate/Vitamin B12” radioassay kit. Detailed laboratory methods and quality control/quality assurance data are available on the NHANES website. Values below the limit of detection (LOD) were replaced with a value of the LOD divided by the square root of 2; *cis*-BC and RS were excluded because 44.6% and 82.9% of their concentrations were under LODs, respectively. Vitamin concentrations were divided into four levels, according to their quartile concentrations.

### 2.3. Assessment of Covariates

Potential confounding variables related to the level of vitamins and depression were examined as follows: gender, BMI, family income to poverty ratio (PIR, a ratio of family income to poverty threshold, the higher the better), race/ethnicity, education, marital status, smoking history, age, and examination time from NHANES websites, demographics, examinations, laboratories and questionnaire data parts. Gender was categorized as male and female. Subjects were categorized into four BMI groups: underweight, normal weight, overweight, and obese by cut-off point 18.5, 25 and 30. We also categorized PIR (0–5) into four groups: below poverty, low-middle income, high-middle income, and high income by cut-off points 1, 2 and 3. There were five categories in race/ethnicity, which included Mexican American, other Hispanic, non-Hispanic White, non-Hispanic Black and other race. Education was divided into three levels: less than high school, high school and greater than high school. Marital status, smoking history and alcohol were categorized into two levels: yes and no. Three categories in age were 20–39, 40–59, and 60–85. Examination time was classified as 1 November through 30 April and 1 May through 31 October.

### 2.4. Assessment of Depression

Depression was assessed by the 9-item Patient Health Questionnaire (PHQ-9), and every question was scored from “0” (not at all) to “3” (nearly every day). Total score of nine PHQ-9 items was dichotomized depression by the cut-off point 10. Depression was defined as a total score no less than 10 [11].

### 2.5. Statistical Analysis

Statistical analysis was performed with Statistical Analysis System statistical software package version 9.2 (SAS Institute Inc., Cary, NC, USA). *p* < 0.05 with two-tailed was considered statistical significance. The different parts data were combined by the respondent sequence number. Number and percent was used to describe categorical variables, including BMI, family PIR, race/ethnicity, education, marital status, smoking history, alcohol age and examination time. Univariate analysis was applied to examine the distribution of vitamin concentrations. Spearman rank-order correlation coefficients were calculated to measure the relationship among vitamin concentrations due to skewed distributions. Binary logistic regression model was used to explore the odds rates (OR) and 95% confidence intervals (CI) between vitamin concentrations and depression in the whole and sex-specific differences. The lowest quartile (Q1) of vitamins was used as reference for second quartile concentrations (Q2), third quartile concentrations (Q3) and the highest quartile concentrations (Q4) [12].

## 3. Results

### 3.1. Baseline Characteristics of All Subjects

In our study population, the prevalence rate of depression was 6.1%, among which the prevalence of male depression accounted for 4.8%, while prevalence in females was represented as 7.4%. Table 1 demonstrated the baseline characteristics of gender-specific population from 20–85 years from NHANES 2005–2006. Age, family PIR and BMI were 49.3 ± 18.2, 2.8 ± 1.6, 28.4 ± 5.6 in males and 49.6 ± 17.8, 2.7 ± 1.6, 29.1 ± 7.3 in females (mean ± standard deviation (SD)), respectively.

### 3.2. Distribution of Serum Vitamin Concentrations

The distribution of serum levels of vitamins (VB9, VB12, PA, PLP, VC, 25(OH)D, AC, *trans*-BC, *cis*-BC, BC, GT, LZ, *trans*-L, RP, RS, VA, AT and TL) were skewed. The detection rates of AC, *trans*-BC, *cis*-BC, BC, LZ, *trans*-L, RP, RS, VA and TL were 91.8%, 99.8%, 55.39%, 99.8%, 99.9%, 99.96%, 78.1%, 17.1%, 99.96% and 99.96%, respectively (Table 2), and the levels of the other kinds of vitamins were all beyond LODs. The detailed distributions of serum vitamin concentrations is shown in Table 2. The Spearman correlation coefficients between *trans*-L and TL concentrations; and the correlation coefficients between *trans*-BC and *cis*-BC concentrations were both greater than 0.9 (Table 3). TL and *trans*-BC concentrations were chosen as representative variants to reduce collinearity of model after principal components analysis. Spearman correlation coefficients showed weak associations among other vitamin concentrations (Table 3).

### 3.3. The Associations between Serum Vitamin Concentrations and Depression

As shown in Table 4, compared to Q1 (function as control group), negative associations were observed among folate concentrations, *trans*-BC concentrations, (*p* for trends were 0.02 and 0.01, respectively) and depression, while positive associations were found among VB12 concentrations, and VA concentrations (*p* for the trends were 0.008 and 0.01, respectively) and depression adjusted by gender, BMI, family PIR, race/ethnicity, education, marital status, smoking history, age and examination time (Table 4). The adjusted ORs (95% CI) for depression were 0.53 (0.33, 0.84) (*p* = 0.007) and 0.47 (0.25, 0.86) (*p* = 0.02) for Q2 and Q4 of Folate, 0.36 (0.16, 0.83) (*p* = 0.02) for the Q4 of *trans*-BC, 1.89 (1.15, 3.11) (*p* = 0.01) for the Q4 of VB12, 2.27 (1.36, 3.81) (*p* = 0.002) for the Q4 of VA compared to their Q1 (Table 4).

### 3.4. Influence of Gender on the Relationship between Serum Vitamin Levels and Depression

Considering physical and psychological differences between males and females, gender difference was taken into account for subgroup analysis. Stratified by gender, we further analyzed the associations between serum vitamin concentrations and depression. As shown in Table 5, in females, folate concentrations was negatively related to depression (*p* for trend = 0.03), while VB12 concentrations was positive correlated with depression (*p* for trend = 0.02) adjusted by BMI, family PIR, race/ethnicity, education, marital status, smoking history, age and examination time. The adjusted ORs (95% CI) for depression were 0.43 (0.23, 0.82) (*p* = 0.01) and 0.38 (0.17, 0.86) (*p* = 0.02) for the Q2 and Q4 of folate concentrations, 2.75 (1.36, 5.59) (*p* = 0.005) for the Q4 of VB12 concentrations compared to the Q1 group. In males, we did not find any significant association between depression and serum vitamin levels.

## 4. Discussion

This cross-sectional study is aimed at evaluating the associations between serum vitamin levels and depression. Previous studies have mainly focused on the relationships between representative vitamin concentrations or specified age groups and depression [13,14,15,16,17,18,19,20]. In this study, we comprehensively investigated the associations between serum vitamin concentrations and depression using sex-specific logistic regression in NHANES 2005–2006. Consistent with previous results, the prevalence of depression was higher in females than that in males (7.4% versus 4.5%). The sex-specific difference was considered for physical and psychological differences between males and females. In the whole populations, serum *trans*-BC and folate concentrations showed inverse associations with depression, while serum VB12 and VA concentrations were positively associated with depression adjusted by gender, BMI, family PIR, race/ethnicity, education, marital status, smoking history, age and examination time. The same associations of folate and VB12 concentrations with depression were found in females, adjusted by BMI, family PIR, race/ethnicity, education, marital status, smoking history, age and examination time.

The result of a case-control study that involved 60 male students was consistent with our research that depression students consumed a lower beta carotene diet [21]. The Kim NR study has also suggested that beta carotene could treat depression [22]. *Trans*-BC, which is mainly absorbed from vegetables and fruits, is an antioxidant vitamin and precursor to VA. The brain is vulnerable to oxidative damage from high consumption of oxygen. Oxidative damage causes the reduction of neurotransmitters, which are associated with the presence of depression [23]. Depression has established a relationship with oxidative stress [24]. Beta carotene improves the brain’s antioxidant states.

Previous literature was inconsistent in associating folate levels and depression. Folate concentrations were unrelated to depression in some studies [19,25]. However, other studies have indicated that the relationship existed. Serum folate concentrations were positively related to depression in reproductive age U.S. women in 2011–2012 NHANES [26]. A low level of folate was reported to be negatively related to depression in the GUSTO study and in other research [27,28,29,30]. Folate supplementation in pregnancy reduced the risk of postpartum depression in a Chinese cohort [31]. In our study, folate concentrations had a negative relationship with depression in the whole population and in females. Folate, also named Vitamin B9, helps to repair DNA damage and produce red blood cells [32]. It provides a methyl group to synthesize methionine, which is the immediate precursor of S-adenosylmethionine (SAM) [33]. SAM is the methyl donor in innumerable methylation reactions in the brain and affects neurotransmitters (such as serotonin, dopamine, noradrenaline) and hormone metabolism [34,35]. Depression is associated with neurotransmitters and hormone disturbances in several biological researches [36,37,38]. A low level of folate and VB12 contribute to hyperhomocysteinemia, which correlates to the risk of depression [20]. Hence, we speculated that a low level of folate was associated with depression.

Folate is also vital to pregnancy for the reason that its deficiency causes birth defects [39]. Women take vitamin supplements to enable them to get essential nutrients required for a healthy pregnancy [40]. Considering this reason, pregnant females were not taken into account for our study.

However, we were surprised that high serum concentrations of VA and VB12 increased the risk of depression. VA is absorbed from animal food, and is vital for growth and development, and necessary for the brain, especially the hippocampus for emotions, learning and memory [41]. Strangely, VA had a positive relationship with depression. For subgroup analysis of gender-specific difference in our study, the association between VA, *trans*-BC concentrations, and depression were not found.

VB12 (cobalamin) also takes part in single carbon transfer methylation, which is vital for lipid, carbohydrate and amino acid metabolism [42]. Consequently, VB12 has a similar function as folate, and enables normal neuronal network function [43]. In previous research, VB12 concentrations had always been reported to have an inverse correlation or no association with depression [25,44,45]. However, VB12 concentrations was positively associated with depression in our study. Only one literature has supported our result that depression was positively correlated with VB12 concentrations in 115 outpatients [46]. Our research was the second to report the positive correlation between serum VB12 concentrations and depression in the high-exposure group. In our research, the P_75_ of serum VB12 concentrations was 482.65 pmol/L. 9.5% in the whole population; 265 participants (170 females and 95 males) were found to be at the higher end (664.2 pmol/L) of the normal range (147.6–664.2 pmol/L) [47]. 37.9% of the participants showed high exposure in VB12 concentrations in Q4 group. VB12 in excessive accumulation led to toxicity in serum. More investigations are needed for further verification.

For other vitamins, PLP, a cofactor form of VB6, contributes to 4% intracellular enzymatic activity by regulating the enzyme cystathionine β-synthase, deficiency of which leads to high plasma total homocysteine [48]. 4-PA is a degradation product of PLP [48]. Both of them have similar functions in one-carbon metabolism as folate and VB12 [49]. VC is a cofactor for the ferrous and 2-oxoglutarate dependent dioxygenases in collagen synthesis and also modulates vasorelaxation [50]. AC, BC, β-cryptoxanthin, TL, LZ are carotenoids absorbed from fruits and vegetables [51]. RP is a retinyl ester, and GT and AT are two species of vitamin E. 25(OH)D is the storage form of vitamin D, which is related to the regulation of the hypothalamic-pituitary-adrenal axis through vitamin D receptors, affecting depression [52]. Absorption of 25(OH)D is mainly affected by ultraviolet B sunlight exposure [53]. Since we have limited authority to access data to acquire an accurate date of examination, we adjusted the two periods of examination (1 November through 30 April and 1 May through 31 October) to reduce seasons’ influence.

In our study, there was no association between 25(OH)D concentrations and depression. However, 25(OH)D concentrations had a negative relationship with depression in some researches [52]. Ultraviolet B sunlight exposure, seasons, and diet were not considered [53,54,55], which largely affect the absorption of 25(OH)D.

There are some disadvantages in our study. For VB12, our study only analyzed the relationship between current serum concentrations and depression, which may cause biological flaws and lead to the wrong conclusions. We also ignored dietary factors, which have an impact on serum vitamins concentrations. Depression is a disease that usually lasts for a prolonged period. It is only possible to estimate prevalence instead of incidence in a cross-sectional study. Prevalence overestimates the incidence rate in depression. Serum vitamins concentrations can be evaluated as a clue to the etiology of depression, and causal relationships between them are unknown in our study.

## 5. Conclusions

Our study mainly investigated the association between serum vitamin levels and depression. The results showed that folate and *trans*-β-carotene concentrations were inversely correlated with depression, while vitamin B12 and Vitamin A concentrations were positively related to depression in the whole populations. Consistent with the results in the whole population, folate and vitamin B12 concentrations were also identified to be correlated with depression in the females. The correlations of vitamin levels with depression deserve further investigation in larger and diverse populations in prospective studies or randomized controlled trials, especially in females.

## Figures and Tables

**Table 1:** Baseline characteristics of participants with depression or no depression in different gender participants aged 20–85 in NHANES 2005–2006 (*n* = 2791).

Characteristic (2791)	Male (1443)		*p*-Value	Female (1348)		*p*-Value
	No Depression	Depression		No Depression	Depression	
*n*	1374 (95.2)	69 (4.8)		1247 (92.6)	101 (7.4)	
Body Mass Index (kg/m^2^)			0.02			0.30
Underweight (<18.5) (52)	20 (1.5)	1 (1.4)		30 (2.4)	1 (1.0)	
Normal weight (18.5-24.9) (795)	348 (25.3)	18 (26.1)		401 (32.2)	28 (27.7)	
Overweight (25.0-29.9) (972)	584 (42.5)	18 (26.1)		345 (27.7)	25 (24.7)	
Obese (≥30.0) (972)	422 (30.7)	32 (46,4)		471 (37.8)	47 (46.5)	
Family PIR			0.004			<0.001
<1.0 (below poverty) (456)	205 (14.9)	17 (24.6)		200 (16.0)	34 (33.7)	
1.0-1.9 (low-middle income) (675)	313 (22.8)	22 (31.9)		311 (24.9)	29 (28.7)	
2.0-2.9 (high-middle income) (435)	228 (16.6)	13 (18.8)		178 (14.3)	16 (15.8)	
≥3.0 (high income) (1225)	628 (45.7)	17 (24.6)		558 (44.7)	22 (21.8)	
Race-ethnicity			0.19			0.10
Mexican American (544)	271 (19.7)	12 (17.4)		246 (19.7)	15 (14.9)	
Other Hispanic (87)	44 (3.2)	1 (1.4)		38 (3.0)	4 (4.0)	
Non-Hispanic White (1438)	729 (53.1)	31 (44.9)		634 (50.8)	44 (43.6)	
Non-Hispanic Black (612)	285 (20.7)	23 (33.3)		270 (21.7)	34 (33.7)	
Other Race (110)	45 (3.3)	2 (2.9)		59 (4.7)	4 (4.0)	
Education			0.03			0.01
<High School (707)	378 (27.5)	23 (33.3)		277 (22.2)	29 (28.7)	
High school (679)	324 (23.6)	23 (33.3)		299 (24.0)	33 (32.7)	
>High school (1405)	672 (48.9)	23 (33.3)		671 (53.8)	39 (38.6)	
Marital status			0.003			0.04
Yes (1549)	841 (61.2)	30 (43.5)		637 (51.1)	41 (40.6)	
No (1242)	533 (38.8)	39 (56.5)		610 (48.9)	60 (59.4)	
Smoking history			0.007			<0.001
Yes (1375)	790 (57.5)	51 (73.9)		478 (38.3)	56 (55.4)	
No (1416)	584 (42.5)	18 (26.1)		769 (61.7)	45 (44.6)	
Alcohol			0.26			0.47
Yes (1974)	1121 (81.6)	60 (87.0)		737 (59.1)	56 (55.4)	
No (817)	253 (18.4)	9 (13.0)		510 (40.9)	45 (44.6)	
Age			0.16			0.01
20–39 (923)	479 (34.8)	19 (27.5)		398 (31.9)	27 (26.7)	
40–59 (960)	448 (32.6)	30 (43.5)		432 (34.6)	50 (49.5)	
60–85 (908)	447 (32.5)	20 (29.0)		417 (33.4)	24 (23.8)	
Examination time			0.65			0.052
1 November through 30 April	616 (44.8)	29 (42.0)		530 (42.5)	53 (52.5)	
1 May through 31 October	758 (55.2)	40 (58.0)		717 (57.5)	48 (47.5)	

Value are number (percent). Smoking history was defined as at least 100 cigarettes in life.

**Table 2:** Distribution of serum vitamin concentrations in participants aged 20–85 in NHANES 2005–2006.

Vitamin	Detection Ratio (%)	Minimun	P10	P25	P50	P75	P90	Maximum
Folate (nmol/L)	100%	1.6	14	19	26	37	51	3.0 × 10^2^
Vitamin B12 (pmol/L)	100%	37	2.0 × 10^2^	2.6 × 10^2^	3.5 × 10^2^	4.8 × 10^2^	6.5 × 10^2^	1.9 × 10^4^
4-pyridoxic acid (nmol/L)	100%	4.6	12	16	26	54	1.4 × 10^2^	1.9 × 10^4^
Pyridoxal 5′-phosphate (nmol/L)	100%	2.0	17	27	44	80	1.6 × 10^2^	1.7 × 10^3^
Vitamin C (umol/L)	100%	0.60	17	36	55	70	88	2.7 × 10^2^
25-hydroxyvitamin D (nmol/L)	100%	13	30	42	57	69	81	2.0 × 10^2^
α-carotene (umol/L)	91.8%	0.0090	0.015	0.028	0.054	0.10	0.19	1.8
*trans*-β-carotene (umol/L)	99.8%	0.011	0.078	0.13	0.23	0.43	0.76	6.2
*cis*-β-carotene (umol/L)	55.4%	0.0090	0.0090	0.0090	0.015	0.026	0.045	0.41
b-Cryptoxanthin (umol/L)	99.8%	0.011	0.058	0.091	0.15	0.25	0.39	1.9
γ-Tocopherol (umol/L)	100%	0.63	2.2	3.3	4.9	6.8	9.2	46
Lutein and zeaxanthin (umol/L)	99.9%	0.030	0.14	0.19	0.27	0.37	0.51	2.0
*trans*-Lycopene (umol/L)	99.96%	0.011	0.18	0.28	0.40	0.55	0.71	1.5
Retinyl Palmitate (umol/L)	78.1%	0.031	0.031	0.049	0.073	0.11	0.16	0.93
Retinyl Stearate (umol/L)	17.1%	0.017	0.017	0.017	0.017	0.017	0.035	0.29
Vitamin A (umol/L)	99.96%	0.024	1.4	1.7	2.0	2.4	2.9	6.5
α-tocopherol (umol/L)	100%	7.2	17	21	26	34	44	1.4 × 10^2^
Total (*cis*- and *trans*-) Lycopene (umol/L)	99.96%	0.013	0.35	0.53	0.75	1.0	1.3	2.8

P10, P25, P50, P75 and P90 represent 10th, 25th, 50th, 75th and 90th percentiles of the serum vitamin concentrations, respectively.

**Table 3:** Correlation matrix among vitamins.

	VB9	VB12	PA	PLP	VC	VD	AC	trans-BC	cis-BC	BC	GT	LZ	trans-L	RP	RS	VA	AT	TL
VB9	1	0.11	0.23	0.29	0.36	0.21	0.12	0.25	0.24	0.06	−0.24	0.08	−0.03	0.27	0.29	0.25	0.34	−0.01
VB12		1	0.05	0.12	0.06	0.04	0.04	0.08	0.07	0.09	−0.08	0.05	−0.02	0.03	0.03	−0.01	0.07	−0.01
PA			1	0.32	0.15	0.04	0.02	0.07	0.07	0.05	−0.09	0.06	−0.02	0.15	0.16	0.18	0.13	<0.01
PLP				1	0.33	0.19	0.15	0.23	0.22	0.13	−0.24	0.14	0.06	0.23	0.20	0.21	0.29	0.09
VC					1	0.20	0.27	0.38	0.36	0.31	−0.34	0.27	0.01	0.26	0.22	0.14	0.32	0.03
VD						1	0.11	0.15	0.14	<0.01	−0.23	0.03	0.07	0.15	0.11	0.26	0.18	0.08
AC							1	0.67	0.65	0.34	−0.19	0.38	0.15	0.29	0.06	0.03	0.16	0.20
*trans*-BC								1	0.98	0.38	−0.26	0.41	0.17	0.31	0.11	0.06	0.29	0.22
*cis*-BC									1	0.35	−0.24	0.41	0.15	0.29	0.10	0.05	0.28	0.20
BC										1	−0.12	0.40	0.16	0.17	<0.01	<0.01	0.14	0.20
GT											1	−0.06	0.08	0.00	−0.01	−0.07	−0.03	0.05
LZ												1	0.19	0.26	0.08	0.11	0.30	0.23
*trans*-L													1	0.38	0.02	0.07	0.15	0.97
RP														1	0.76	0.22	0.43	0.42
RS															1	0.19	0.40	0.03
VA																1	0.37	0.08
AT																	1	0.16
TL																		1

VB9: Folate; VB12: Vitamin B12; PA: 4-pyridoxic acid; PLP: Pyridoxal 5′-phosphate; VC: Vitamin C; 25OHD: 25-hydroxyvitamin D; AC: α-carotene; *trans*-BC: *trans*-β-carotene; *cis*-BC: *cis*-β-carotene; BC: b-Cryptoxanthin; GT: γ-Tocopherol; LZ: Lutein and zeaxanthin; *trans*-L: *trans*-Lycopene; RP: Retinyl Palmitate; RS: Retinyl Stearate; VA: Vitamin A; AT: α-Tocopherol; TL: Total (*cis*- and *trans*-) Lycopene.

**Table 4:** Binary logistic regression crude and adjusted OR (95% CI) (adjusted for gender, BMI, family PIR race/ethnicity, education, marital status smoking history, age and examination time) associations between depression and quartiles of vitamin concentrations.

Exposure		Crude OR (95% CI)	*p*-Value	Adjusted OR (95% CI)	*p*-Value
Folate	Q1	Reference		Reference	
	Q2	0.57 (0.36, 0.89)	0.01	0.53 (0.33, 0.84)	0.007
	Q3	0.74 (0.48, 1.17)	0.21	0.69 (0.43, 1.11)	0.13
	Q4	0.53 (0.30, 0.96)	0.04	0.47 (0.25, 0.86)	0.02
	*p* for trend		0.047		0.02
Vitamin B12	Q1	Reference		Reference	
	Q2	1.43 (0.92, 2.22)	0.11	1.38 (0.87, 2.17)	0.17
	Q3	0.98 (0.59, 1.60)	0.92	0.84 (0.50, 1.41)	0.51
	Q4	2.17 (1.36, 3.48)	0.001	1.89 (1.15, 3.11)	0.01
	*p* for trend		0.002		0.008
4-pyridoxic acid	Q1	Reference		Reference	
	Q2	1.27 (0.81, 1.99)	0.30	1.47 (0.92, 2.36)	0.11
	Q3	1.18 (0.70, 1.99)	0.54	1.36 (0.78, 2.36)	0.28
	Q4	1.18 (0.57, 2.44)	0.65	1.34 (0.62, 2.89)	0.46
	*p* for trend		0.78		0.45
Pyridoxal 5′-phosphate	Q1	Reference		Reference	
	Q2	0.84 (0.54, 1.29)	0.42	1.01 (0.64, 1.59)	0.98
	Q3	0.67 (0.40, 1.12)	0.13	0.90 (0.51, 1.56)	0.70
	Q4	0.66 (0.34, 1.29)	0.22	0.94 (0.45, 1.96)	0.87
	*p* for trend		0.45		0.98
Vitamin C	Q1	Reference		Reference	
	Q2	0.82 (0.52, 1.28)	0.38	0.83 (0.52, 1.33)	0.44
	Q3	1.10 (0.69, 1.76)	0.70	1.15 (0.69, 1.89)	0.60
	Q4	0.68 (0.38, 1.23)	0.20	0.61 (0.33, 1.12)	0.11
	*p* for trend		0.29		0.14
25-hydroxyvitamin D	Q1	Reference		Reference	
	Q2	0.89 (0.58, 1.36)	0.57	1.05 (0.66, 1.66)	0.85
	Q3	0.78 (0.48, 1.26)	0.31	1.05 (0.61, 1.78)	0.87
	Q4	0.58 (0.36, 0.95)	0.03	0.81 (0.46, 1.43)	0.47
	*p* for trend		0.18		0.76
α-carotene	Q1	Reference		Reference	
	Q2	0.85 (0.54, 1.34)	0.48	0.87 (0.54, 1.41)	0.57
	Q3	0.93 (0.54, 1.60)	0.79	1.03 (0.58, 1.82)	0.93
	Q4	1.13 (0.57, 2.21)	0.73	1.20 (0.59, 2.45)	0.62
	*p* for trend		0.77		0.78
*trans*-β-carotene	Q1	Reference		Reference	
	Q2	1.32 (0.84, 2.07)	0.23	1.21 (0.76, 1.93)	0.42
	Q3	0.99 (0.553, 1.76)	0.96	0.81 (0.45, 1.47)	0.49
	Q4	0.53 (0.24, 1.16)	0.11	0.36 (0.16, 0.83)	0.02
	*p* for trend		0.06		0.01
b-Cryptoxanthin	Q1	Reference		Reference	
	Q2	0.88 (0.56, 1.38)	0.58	0.88 (0.55, 1.41)	0.59
	Q3	0.71 (0.42, 1.22)	0.22	0.81 (0.45, 1.44)	0.47
	Q4	0.90 (0.49, 1.64)	0.72	1.10 (0.56, 2.16)	0.78
	*p* for trend		0.64		0.68
γ-Tocopherol	Q1	Reference		Reference	
	Q2	1.15 (0.69, 1.91)	0.60	1.05 (0.62, 1.77)	0.87
	Q3	0.98 (0.58, 1.66)	0.94	0.84 (0.49, 1.46)	0.54
	Q4	1.15 (0.68, 1.95)	0.61	0.87 (0.50, 1.52)	0.62
	*p* for trend		0.86		0.80
Lutein and zeaxanthin	Q1	Reference		Reference	
	Q2	0.65 (0.42, 1.00)	0.050	0.60 (0.38, 0.96)	0.03
	Q3	0.61 (0.37, 1.00)	0.049	0.63 (0.37, 1.07)	0.09
	Q4	0.54 (0.30, 0.96)	0.04	0.58 (0.31, 1.08)	0.09
	*p* for trend		0.01		0.14
Retinyl Palmitate	Q1	Reference		Reference	
	Q2	0.92 (0.59, 1.45)	0.72	0.95 (0.60, 1.53)	0.84
	Q3	0.91 (0.54, 1.54)	0.73	1.00 (0.58, 1.73)	0.99
	Q4	0.79 (0.43, 1.45)	0.45	0.90 (0.47, 1.70)	0.74
	*p* for trend		0.90		0.98
Vitamin A	Q1	Reference		Reference	
	Q2	1.07 (0.66, 1.73)	0.78	1.22 (0.74, 2.01)	0.43
	Q3	1.33 (0.81, 2.16)	0.26	1.52 (0.92, 2.51)	0.10
	Q4	2.03 (1.23, 3.33)	0.005	2.27 (1.36, 3.81)	0.002
	*p* for trend		0.02		0.01
α-Tocopherol	Q1	Reference		Reference	
	Q2	0.83 (0.53, 1.29)	0.40	0.86 (0.54, 1.38)	0.53
	Q3	0.82 (0.50, 1.34)	0.43	0.86 (0.51, 1.47)	0.58
	Q4	1.07 (0.61, 1.88)	0.82	1.13 (0.61, 2.10)	0.69
	*p* for trend		0.63		0.69
Total (*cis*- and *trans*-) Lycopene	Q1	Reference		Reference	
	Q2	0.88 (0.57, 1.38)	0.58	0.90 (0.57, 1.42)	0.64
	Q3	0.87 (0.53, 1.42)	0.57	0.88 (0.52, 1.48)	0.62
	Q4	0.93 (0.52, 1.64)	0.79	0.96 (0.52, 1.77)	0.89
	*p* for trend		0.93		0.94

Folate (nmol/L): Q1 ≤ 18.8; Q2: 18.8–26.3; Q3: 26.3–36.9; Q4 > 36.9. Vitamin B12 (pmol/L): Q1 ≤ 260.51; Q2: 260.51–354.24; Q3: 354.24–482.65; Q4 > 482.65. 4-pyridoxic acid (nmol/L): Q1 ≤ 16.2; Q2: 16.2–26.1; Q3: 26.1–54.0; Q4 > 54.0. Pyridoxal 5′-phosphate (nmol/L): Q1 ≤ 26.7; Q2: 26.7–43.7; Q3: 43.7–79.9; Q4 > 79.9. Vitamin C (umol/L): Q1 ≤ 36.3; Q2: 36.3–54.5; Q3: 54.5–69.8; Q4 > 69.8. 25-hydroxyvitamin D (nmol/L): Q1 ≤ 42.2; Q2: 42.2–56.8; Q3: 56.8–68.9; Q4 > 68.9. α-carotene (umol/L): Q1 ≤ 0.028; Q2: 0.028–0.054; Q3: 0.054–0.102; Q4 > 0.102. *trans*-β carotene (umol/L): Q1 ≤ 0.130; Q2: 0.130–0.233; Q3: 0.233–0.427; Q4 > 0.427. b-Cryptoxanthin (umol/L): Q1 ≤ 0.091; Q2: 0.091–0.150; Q3: 0.150–0.248; Q4 > 0.248. γ-Tocopherol (umol/L): Q1 ≤ 3.339; Q2: 3.339–4.924; Q3: 4.924–6.822; Q4 > 6.822. Lutein and zeaxanthin (umol/L): Q1 ≤ 0.186; Q2: 0.186–0.267; Q3: 0.267–0.369; Q4 > 0.369. Retinyl Palmitate (umol/L): Q1 ≤ 0.049; Q2: 0.049–0.073; Q3: 0.073–0.108; Q4 > 0.108. Vitamin A (umol/L): Q1 ≤ 1.676; Q2: 1.676–2.011; Q3: 2.011–2.419; Q4 > 2.419. α-Tocopherol (umol/L): Q1 ≤ 20.991; Q2: 20.991–26.239; Q3: 26.239–33.669; Q4 > 33.669. Total (*cis*- and *trans*-) Lycopene (umol/L): Q1 ≤ 0.527; Q2: 0.527–0.751; Q3: 0.751–1.019; Q4 > 1.019.

**Table 5:** Binary logistic regression OR (95% CI) (adjusted for BMI, family PIR race/ethnicity, education, marital status smoking history, age and examination time) associations between depression and quartiles of vitamin concentrations in different genders.

Exposure		Male OR (95% CI)	*p*-Value	Female OR (95% CI)	*p*-Value
Folate	Q1	Reference		Reference	
	Q2	0.60 (0.29, 1.26)	0.18	0.43 (0.23, 0.82)	0.01
	Q3	0.72 (0.34, 1.55)	0.40	0.64 (0.34, 1.20)	0.17
	Q4	0.53 (0.19, 1.44)	0.21	0.38 (0.17, 0.86)	0.02
	*p* for trend		0.48		0.03
Vitamin B12	Q1	Reference		Reference	
	Q2	1.31 (0.64, 2.67)	0.46	1.67 (0.88, 3.16)	0.11
	Q3	0.64 (0.28, 1.48)	0.29	1.17 (0.58, 2.37)	0.67
	Q4	1.29 (0.59, 2.82)	0.53	2.75 (1.36, 5.59)	0.005
	*p* for trend		0.28		0.02
4-pyridoxic acid	Q1	Reference		Reference	
	Q2	2.47 (1.00, 6.10)	0.049	1.43 (0.78, 2.62)	0.25
	Q3	2.67 (0.99, 7.16)	0.052	1.21 (0.58, 2.55)	0.62
	Q4	2.41 (0.67, 8.66)	0.18	1.17 (0.38, 3.56)	0.79
	*p* for trend		0.21		0.72
Pyridoxal 5′-phosphate	Q1	Reference		Reference	
	Q2	1.09 (0.49, 2.40)	0.83	0.84 (0.46, 1.54)	0.57
	Q3	0.77 (0.32, 1.89)	0.57	0.91 (0.42, 1.97)	0.81
	Q4	0.81 (0.27, 2.49)	0.72	0.92 (0.30, 2.82)	0.88
	*p* for trend		0.84		0.96
Vitamin C	Q1	Reference		Reference	
	Q2	0.85 (0.40, 1.80)	0.67	0.80 (0.42, 1.53)	0.49
	Q3	1.52 (0.69, 3.34)	0.30	0.97 (0.49, 1.93)	0.93
	Q4	0.77 (0.28, 2.06)	0.60	0.53 (0.23, 1.19)	0.12
	*p* for trend		0.35		0.37
25-hydroxyvitamin D	Q1	Reference		Reference	
	Q2	0.64 (0.28, 1.47)	0.29	1.36 (0.75, 2.47)	0.32
	Q3	1.23 (0.54, 2.81)	0.62	0.91 (0.42, 1.98)	0.81
	Q4	0.79 (0.33, 1.92)	0.60	0.77 (0.35, 1.72)	0.53
	*p* for trend		0.38		0.44
α-carotene	Q1	Reference		Reference	
	Q2	0.70 (0.33, 1.47)	0.34	0.95 (0.49, 1.87)	0.88
	Q3	0.65 (0.25, 1.69)	0.37	1.36 (0.62, 2.99)	0.44
	Q4	0.81 (0.24, 2.68)	0.72	1.39 (0.54, 3.61)	0.50
	*p* for trend		0.75		0.74
*trans*-β-carotene	Q1	Reference		Reference	
	Q2	1.33 (0.66, 2.70)	0.42	0.99 (0.51, 1.93)	0.98
	Q3	0.75 (0.29, 1.95)	0.56	0.87 (0.38, 1.97)	0.73
	Q4	0.20 (0.04, 0.99)	0.048	0.57 (0.19, 1.67)	0.30
	*p* for trend		0.08		0.70
b-Cryptoxanthin	Q1	Reference		Reference	
	Q2	1.12 (0.52, 2.42)	0.77	0.72 (0.38, 1.37)	0.31
	Q3	1.15 (0.47, 2.85)	0.76	0.55 (0.25, 1.23)	0.14
	Q4	2.04 (0.73, 5.66)	0.17	0.57 (0.22, 1.47)	0.24
	*p* for trend		0.51		0.51
γ-Tocopherol	Q1	Reference		Reference	
	Q2	0.71 (0.32, 1.58)	0.40	1.25 (0.59, 2.65)	0.56
	Q3	0.52 (0.22, 1.23)	0.14	1.07 (0.49, 2.32)	0.87
	Q4	0.51 (0.21, 1.23)	0.13	1.10 (0.49, 2.46)	0.81
	*p* for trend		0.41		0.94
Lutein and zeaxanthin	Q1	Reference		Reference	
	Q2	0.53 (0.25, 1.13)	0.10	0.67 (0.36, 1.28)	0.22
	Q3	0.63 (0.27, 1.45)	0.27	0.64 (0.30, 1.37)	0.25
	Q4	0.46 (0.17, 1.22)	0.12	0.70 (0.29, 1.69)	0.43
	*p* for trend		0.32		0.60
Retinyl Palmitate	Q1	Reference		Reference	
	Q2	1.09 (0.49, 2.46)	0.82	0.75 (0.41, 1.40)	0.37
	Q3	1.49 (0.62, 3.59)	0.37	0.57 (0.27, 1.22)	0.15
	Q4	1.74 (0.67, 4.49)	0.25	0.32 (0.12, 0.88)	0.03
	*p* for trend		0.64		0.18
Vitamin A	Q1	Reference		Reference	
	Q2	1.57 (0.63, 3.92)	0.33	1.10 (0.58, 2.10)	0.76
	Q3	1.38 (0.53, 3.59)	0.51	1.72 (0.91, 3.26)	0.10
	Q4	2.77 (1.10, 6.94)	0.03	2.18 (1.08, 4.40)	0.03
	*p* for trend		0.10		0.11
α-Tocopherol	Q1	Reference		Reference	
	Q2	1.03 (0.47, 2.27)	0.94	0.69 (0.37, 1.31)	0.26
	Q3	1.01 (0.41, 2.50)	0.98	0.68 (0.33, 1.40)	0.30
	Q4	1.46 (0.54, 3.95)	0.46	0.84 (0.36, 1.97)	0.69
	*p* for trend		0.82		0.64
Total (*cis*- and *trans*-)Lycopene	Q1	Reference		Reference	
	Q2	1.23 (0.59, 2.57)	0.58	0.70 (0.37, 1.34)	0.28
	Q3	0.67 (0.28, 1.61)	0.37	1.42 (0.70, 2.88)	0.33
	Q4	0.69 (0.27, 1.77)	0.44	1.95 (0.82, 4.64)	0.13
	*p* for trend		0.41		0.11

Folate (nmol/L): Q1 ≤ 18.8; Q2: 18.8–26.3; Q3: 26.3–36.9; Q4 > 36.9. Vitamin B12 (pmol/L): Q1 ≤ 260.51; Q2: 260.51–354.24; Q3: 354.24–482.65; Q4 > 482.65. 4-pyridoxic acid (nmol/L): Q1 ≤ 16.2; Q2: 16.2–26.1; Q3: 26.1–54.0; Q4 > 54.0. Pyridoxal 5′-phosphate (nmol/L): Q1 ≤ 26.7; Q2: 26.7–43.7; Q3: 43.7–79.9; Q4 > 79.9. Vitamin C (umol/L): Q1 ≤ 36.3; Q2: 36.3–54.5; Q3: 54.5–69.8; Q4 > 69.8. 25-hydroxyvitamin D (nmol/L): Q1 ≤ 42.2; Q2: 42.2–56.8; Q3: 56.8–68.9; Q4 > 68.9. α-carotene (umol/L): Q1 ≤ 0.028; Q2: 0.028–0.054; Q3: 0.054–0.102; Q4 > 0.102. *trans*-β carotene (umol/L): Q1 ≤ 0.130; Q2: 0.130–0.233; Q3: 0.233–0.427; Q4 > 0.427. b-Cryptoxanthin (umol/L): Q1 ≤ 0.091; Q2: 0.091–0.150; Q3: 0.150–0.248; Q4 > 0.248. γ-Tocopherol (umol/L): Q1 ≤ 3.339; Q2: 3.339–4.924; Q3: 4.924–6.822; Q4 > 6.822. Lutein and zeaxanthin (umol/L): Q1 ≤ 0.186; Q2: 0.186–0.267; Q3: 0.267–0.369; Q4 > 0.369. Retinyl Palmitate (umol/L): Q1 ≤ 0.049; Q2: 0.049–0.073; Q3: 0.073–0.108; Q4 > 0.108. Vitamin A (umol/L): Q1 ≤ 1.676; Q2: 1.676–2.011; Q3: 2.011–2.419; Q4 > 2.419. α-Tocopherol (umol/L): Q1 ≤ 20.991; Q2: 20.991–26.239; Q3: 26.239–33.669; Q4 > 33.669. Total (*cis*- and *trans*-) Lycopene (umol/L): Q1 ≤ 0.527; Q2: 0.527–0.751; Q3: 0.751–1.019; Q4 > 1.019.
